# Antioxidant, Anti-Inflammatory, and Antibacterial Properties of an *Achillea millefolium* L. Extract and Its Fractions Obtained by Supercritical Anti-Solvent Fractionation against *Helicobacter pylori*

**DOI:** 10.3390/antiox11101849

**Published:** 2022-09-20

**Authors:** Marisol Villalva, Jose Manuel Silvan, Teresa Alarcón-Cavero, David Villanueva-Bermejo, Laura Jaime, Susana Santoyo, Adolfo J. Martinez-Rodriguez

**Affiliations:** 1 Microbiology and Food Biocatalysis Group (MICROBIO), Department of Biotechnology and Food Microbiology, Institute of Food Science Research (CIAL, CSIC-UAM), C/Nicolás Cabrera, 9. Cantoblanco Campus, Autonomous University of Madrid, 28049 Madrid, Spain; 2 Microbiology Department, Hospital Universitario de La Princesa, 28006 Madrid, Spain; 3 Department of Preventive Medicine, Public Health and Microbiology, School of Medicine, Autonomous University of Madrid, 28029 Madrid, Spain; 4 Department of Production and Characterization of Novel Foods, Institute of Food Science Research (CIAL, CSIC-UAM), C/Nicolas Cabrera, 9. Cantoblanco Campus, Autonomous University of Madrid, 28049 Madrid, Spain

**Keywords:** *Achillea millefolium*, yarrow extract, *H. pylori*, supercritical anti-solvent fractionation, anti-inflammatory activity, antioxidant activity, antibacterial activity

## Abstract

The main objective of this work is to evaluate the potential utility of an *Achillea millefolium* extract (yarrow extract, YE) in the control of *H. pylori* infection. The supercritical anti-solvent fractionation (SAF) process of YE allowed the obtaining of two different fractions: yarrow’s precipitated fraction (YPF), enriched in most polar phenolic compounds (luteolin-7-*O*-glucoside, luteolin, and 3,5-dicaffeoylquinic acid), and yarrow’s separator fraction (YSF), enriched in monoterpenes and sesquiterpenes, mainly containing camphor, artemisia ketone, and borneol. YE was effective in reducing reactive oxygen species (ROS) production in human gastric AGS cells by 16% to 29%, depending on the *H. pylori* strain. YPF had the highest inhibitory activity (38–40%) for ROS production. YE modulated the inflammatory response in AGS gastric cells, decreasing IL-8 production by 53% to 64%. This IL-8 inhibition also showed a strain-dependent character. YPF and YSF exhibited similar behavior, reducing IL-8 production, suggesting that both phenolic compounds and essential oils could contribute to IL-8 inhibition. YSF showed the highest antibacterial activity against *H. pylori* (6.3–7.1 log CFU reduction, depending on the strain) and lower MIC (0.08 mg/mL). Results obtained have shown that YE and SAF fractions (YPF and YSF) were effective as antioxidant, anti-inflammatory, and antibacterial agents regardless of the *H. pylori* strain characteristics.

## 1. Introduction

*Helicobacter pylori* (*H. pylori*) is one of the most prevalent human pathogens, as over half of the world’s population is colonized with this Gram-negative bacterium [1]. The gastric colonization by *H. pylori* occurs asymptomatically in most individuals, although most people infected with *H. pylori* usually have histological changes in gastric mucosa consistent with the presence of gastritis. However, long-term infection with the pathogen can cause a wide range of clinical manifestations associated with several diseases, including gastric inflammation, peptic ulcer, gastric cancer, gastric mucosa-associated lymphoid-tissue lymphoma, and other extra-gastric pathologies [2]. Due to the high correlation between *H. pylori* infection and gastric cancer, most therapeutic guidelines aim to eradicate this pathogen using a combination of antibiotics with a proton pump inhibitor in triple or quadruple therapy [3]. However, there are a number of concerns related to the use of eradicative therapies, especially in asymptomatic individuals. First, the global increase in antibiotic resistance [4] and the significant distress that antibiotic therapy causes in the microbiota [5]; and second, the relationship that has been found between the use of eradicative therapies and the emergence or worsening of other pathologies, such as esophageal reflux [6]. This situation has led to increased interest in bioactive compounds obtained from natural sources for the treatment of *H. pylori* infection [7]. Natural extracts not only with antibacterial activity against *H. pylori* but also with anti-inflammatory and antioxidant properties could be potentially interesting in *H. pylori* treatment [8,9,10,11]. This is because the immune response to *H. pylori* is a combination of events involving both protective and damaging responses to the host. In fact, it has been described that much of the pathological evidence related to *H. pylori* infection may be due more to the effects of the host’s immune system than to the bacterial infection itself [12].

*Achillea millefolium* L.—traditionally known as yarrow—is a flowering plant commonly used in folk medicine not only in Europe but also in Asia, Africa, and America [13]. Due to the widely known benefits of this plant, the study of its composition and biological properties has awakened a constant interest in developing pharmaceutical, nutraceutical, and food products [14,15,16]. Dried and fresh upper parts from yarrow have been used to prepare aqueous and alcoholic extracts for the treatment of several health problems, such as diabetes and cardiovascular, respiratory, hepatobiliary, spasmodic, and gastrointestinal disorders [17]. In addition, yarrow has been used externally for the treatment of skin and mucous membrane inflammation [18]. The main bioactive compounds present in different yarrow extracts have been associated to health benefits. The presence of phenolic compounds, specifically chlorogenic and dicaffeoylquinic acids, luteolin, apigenin, and quercetin, as well as volatile fraction constituents, predominantly terpenes, such as borneol, camphor, 1,8-cineole, and chamazulene, have been related to antioxidant, anti-inflammatory, antibacterial, antitumor, and antidiabetic properties [19,20,21,22]. The antioxidant effect of yarrow extracts has been extensively studied in both in vitro and in vivo models; likewise, the radical scavenging capacity, intracellular oxidative damage, and reduction in lipid peroxidation in rats have been reported [13,23,24,25,26]. The anti-inflammatory properties of yarrow ethanolic extracts have shown their role in the suppression of pro-inflammatory cytokines [26,27]. Regarding antibacterial activity, aqueous and ethanolic extracts of yarrow have been effective against different microorganisms, including those causing skin infections, such as *Staphylococcus aureus*, *Staphylococcus epidermidis*, and *Pseudomonas aeruginosa*, and others related with gastrointestinal diseases, such as *Salmonella thypi* and *Escherichia coli* [19,28,29]. However, the effect of yarrow on *H. pylori* is scarcely known despite it being one of the main human pathogens. Only two previous studies screening different extracts obtained from plants used in traditional medicine have shown an antibacterial effect of yarrow against *H. pylori* [30,31]. There are no previous reports on the antioxidant and anti-inflammatory effect of yarrow on human gastric cells infected with *H. pylori*.

Since continuously increasing research on bioactive components and rising interest in high-quality ingredients is evident, manufacturers are motivated to use enriched extracts, fractions, or purified components instead of crude extracts. Furthermore, the use of clean and sustainable extract processes is an essential requirement nowadays. For that purpose, different approaches have been explored to obtain fractions enriched in bioactive molecules from plant extracts, mainly phenolic compounds, such as the use of membrane technology [32], solid-phase extraction with reusable macroporous resins [33], and supercritical anti-solvent fractionation (SAF) with CO_2_ at supercritical conditions as a solvent [34,35]. The SAF technique has gained interest as a fractionation or purification process with the potential to reduce the number of steps, since as well as separation of compound(s) occurring in the precipitate, a dried enriched-precipitate is produced [36]. Another advantage of SAF is the low use of chemicals and the reduction in waste that is due to CO_2_ being recycled for further extractions. Recently, we have demonstrated that SAF resulted in an adequate method to improve the antioxidant and anti-inflammatory properties of a yarrow ethanolic extract [37], although its impact against *H. pylori* is unknown. For this reason, in this study, we have evaluated the antioxidant, anti-inflammatory, and antibacterial properties of a yarrow extract and its fractions obtained by SAF against three different *H. pylori* strains.

## 2. Materials and Methods

### 2.1. Sample Material and Ultrasound-Assisted Extraction of Yarrow

Inflorescences and upper dried leaves from yarrow (*Achillea millefollium* L.) were purchased from a local herbalist (Murciana Herbolisteria, Murcia, Spain). The sun-dried plant from a Bulgarian variety was ground in a hammer mill (Premill 250, Lleal S.A., Granollers, Spain) and sieved to reduce its particle size (<500 μm). Then, the UAE extraction was carried out by using an ultrasonic device (Branson Digital Sonifier 250, Danbury, CT, USA) with a power of 200 W and frequencies of 60 kHz. For this purpose, 40 g of ground and sieved yarrow plant were added to 400 mL of pure ethanol (Panreac Madrid, Spain) for 30 min at 40 °C. An output of 70% with respect to the nominal amplitude was applied during extraction. Finally, the obtained yarrow extract (YE) was concentrated to a final concentration of 17.9 mg/mL by rotary evaporation at 35 °C (IKA RV-10 control, VWR, Madrid, Spain) and stored at −20 °C.

### 2.2. Supercritical Anti-Solvent Fractionation (SAF) of Yarrow Extract

Fractionation of YE was performed by means of a piece of supercritical technology equipment (Thar SF2000, Thar Technology, Pittsburgh, PA, USA) with two pumps for the separate supply of supercritical CO_2_ (SC-CO_2_) and YE solution, and a precipitation vessel and two separators’ vessels (0.5 L each), with independent control of temperature and pressure as described by Villanueva-Bermejo et al. [35]. Briefly, SC-CO_2_ was pumped into the precipitation vessel until 15 MPa of pressure and 40 °C were attained. Then, the solution of YE (17.9 mg/mL concentration) was pumped into the precipitator while maintaining the SC-CO_2_ flow. A CO_2_/extract flow ratio of 31.3 g/g (50 g/min for CO_2_ and 1.6 g/min for YE) was employed. During the process, both separators’ vessels were kept at ambient pressure. After system depressurization, two fractions were collected, one corresponding to the YE components that were not soluble in the SC-CO_2_+ethanol mixture and precipitated in the precipitation vessel (yarrow’s precipitated fraction, YPF). The second fraction corresponded to the YE components soluble in the SC-CO_2_+ethanol recovered in the separators (yarrow’s separator fraction, YSF) with an oleoresin appearance. To obtain a dried YSF fraction, the samples of both separator vessels were recovered with ethanol and combined in a single fraction to finally remove the solvent by rotary evaporation under vacuum. The YPF and YSF fractions were kept at −20 °C in darkness until analysis.

### 2.3. Chemical Characterization of YE and Its Fractions by HPLC-PAD-ESI-QTOF-MS and GC-MS Analyses

The phenolic composition was determined by HPLC using an Agilent HPLC 1260 Infinity series system (Agilent Technologies Inc., Santa Clara, CA, USA) according to the Villalva et al. [37] methodology. Chromatographic separation was carried out by using a reverse phase ACE Excell 3 Super C18 column (150 mm × 4.6 mm, 3 μm particle size) from Advanced Chromatography Technologies (Aberdeen, Scotland), thermostated at 35 °C and protected by an ACE 3 C18-AR (10 mm, ×3 mm) guard column. Dry samples were dissolved in DMSO (HPLC grade, ≥99.7%) (Merck, Madrid, Spain) to allow a final concentration of 5 mg/mL and filtered by a PVDF filter (0.45 μm) before injection (20 μL). For identification purposes, the retention time (Rt) and UV–Vis spectrum of each chromatographic peak were compared with the analytical standards (Phytolab, Madrid, Spain); additionally, the accurate mass from HPLC-ESI-QTOF-MS in negative mode analysis was used for compounds assignment, as previously described in Villalva et al. [37]. For quantification, standard calibration curves were built for each pure compound, namely, caffeic acid, caftaric acid, chlorogenic acid, cryptochlorogenic acid, 1,5-dicaffeoylquinic acid (DCQA), 3,4-DCQA, 3,5-DCQA, 4,5-DCQA, ferulic acid, neochlorogenic acid, rosmarinic acid, apigenin, apigenin-7-*O*-glucoside, diosmetin, homoorientin, luteolin-*β*-7-O-glucuronide, luteolin-7-*O*-glucoside, schaftoside, vicenin 2, casticin, quercetin, rutin, and vitexin. Moreover, luteolin-6,8-di-*C*-glucoside and 6-hydroxyluteolin-7-*O*-glucoside were quantified by the calibration curve of orientin and luteolin-7-*O*-glucoside. In addition, vicenin 2 and schaftoside calibration curves were used for apigenin-*C*-hexoside-*C*-pentoside and schaftoside isomer quantification; as well, quercetin and casticin were used for methoxyquercetin isomer and centaureidin, respectively.

Volatile compounds from yarrow extracts were characterized by GC-MS using an Agilent 7890A system (Agilent Technologies, Santa Clara, CA, USA) equipped with a split/splitless auto-injector (G4513A), a flame ionization detector, a triple-axis mass spectrometer detector (5975C), and GC/MS Solution software. Extracts were dissolved in ethanol (5 mg/mL final concentration), filtered (0.45 μm), and injected (1 μL) in splitless mode. Then, the chromatographic analysis was carried out as described by Villalva et al. [37]. Briefly, the mass spectrometer operated under electron impact mode (70 eV) and it was used in total ion current (TIC) mode (mass range from 40 to 500 *m*/*z*). The analysis was performed using an Agilent HP-5MS capillary column (30 m × 0.25 mm i.d., 0.25 μm phase thickness) and the following chromatographic method: 40 °C initial temperature, from 40 °C to 150 °C at 3 °C min^−1^, isothermal at 150 °C for 10 min, then increased from 150 to 300 °C at 6 °C min^−1^, and finally isothermal at 300 °C for 1 min. Helium (99.99%) was employed as the carrier gas (1 mL/min flow rate). The temperature used for the injector was 250 °C. For the identification of volatile compounds, the obtained mass spectral fragmentation patterns were contrasted with those of the Wiley 229 mass spectral library. In addition, their corresponding retention indices were calculated and compared to the information reported in the literature [38,39,40,41] and contained in the NIST database.

### 2.4. Helicobacter pylori, Growth Media, and Culture Conditions

*H. pylori* strains (Hp48, Hp53, and Hp59) were isolated from gastric mucosal biopsies obtained from symptomatic patients from the Microbiology Department, Hospital Universitario La Princesa (Madrid, Spain). Biopsies were cultured in selective (Pylori agar, BioMerieux, Madrid, Spain) and non-selective media (blood-supplemented Columbia Agar, BioMerieux, Madrid, Spain). Hp48 and Hp59 strains are resistant to metronidazole, while Hp53 is a multi-resistant strain with resistance to amoxicillin, clarithromycin, and rifampicin. *H. pylori* strains were stored at −80 °C in Brucella broth (BB) (Becton, Dickinson, & Co., Madrid, Spain) with 20% glycerol. The agar-plating medium consisted of Müeller–Hinton agar supplemented with 5% defibrinated sheep blood (MHB) (Becton, Dickinson, & Co.), and the liquid growth medium consisted of BB supplemented with 10% horse serum (HS) (Biowest, Barcelona, Spain). *H. pylori* inoculum strains were prepared as follows: the frozen stored strains were reactivated by inoculation (200 μL) in a MHB plate and incubation in a microaerophilic atmosphere using a variable atmosphere incubator (VAIN) (85% N_2_, 10% CO_2_, 5% O_2_) (MACS-VA500, Don Whitley Scientific, Bingley, UK) at 37 °C for 72 h. Bacterial biomass grown in one MHB plate was collected with a sterile cotton swab and suspended in 2 mL of BB supplemented with 10% HS (BB-HS) or a culture medium cell (~1 × 10^8^ colony forming units/mL (CFU/mL)), and was used as an experimental bacterial inoculum in the different experimental assays.

### 2.5. Human Gastric Epithelial Cell Cultures

The human gastric epithelial cell line AGS was obtained from the American Type Culture Collection (ATCC, Barcelona, Spain). Cells were grown in Dulbecco’s Modified Eagle’s Medium/F12 (DMEM/F12) (Lonza, Madrid, Spain) supplemented with 10% fetal bovine serum (FBS) of South American origin (Hyclone, GE Healthcare, Logan, UK) and 1% penicillin/streptomycin (5000 U/mL) (Lonza). Cells were plated at densities of ~1 × 10^6^ cells in 75 cm^2^ culture flasks (Sarstedt, Barcelona, Spain) and incubated at 37 °C under 5% CO_2_ in a humidified incubator until 90% confluence was reached. The culture cell medium was changed every two days. Before a confluent monolayer appeared, a cell sub-culturing process was carried out. All experiments were performed between passage 5 and 15 to ensure cell uniformity and reproducibility.

### 2.6. Cell Viability

Before conducting experiments on antioxidant and anti-inflammatory activity, it was necessary to evaluate the potential cytotoxicity of YE and its fractions (YPF and YSF) against the AGS cell line at different concentrations. For this purpose, cell viability was determined by the MTT (3-(4,5-dimethylthiazol-2-yl)-2,5-diphenyltetrazolium bromide) (Merck) reduction assay, as was previously described by Silvan et al. [10]. Confluent cell cultures (~90%) were trypsinized (Trypsin/EDTA solution 170,000 U/L) (Lonza) and cells were seeded (~5 × 10^4^ cells per well) in 96-well plates (Sarstedt) and incubated in cell culture medium at 37 °C under 5% CO_2_ in a humidity incubator for 24 h. Cell culture medium was replaced with a serum-free cell culture medium containing YE and its fractions at 0.4, 0.2, and 0.08 mg/mL (final concentration), and cells were incubated at 37 °C under 5% CO_2_ in a humidity incubator for 24 h. Viability control cells (non-treated) were incubated in a serum-free cell culture medium without samples. Thereafter, cells were washed twice with phosphate-buffered saline (PBS) (Lonza), and the medium was replaced with 200 μL of serum-free cell culture medium plus 20 μL of MTT solution in PBS (5 mg/mL) that were added to each well for the quantification of the living, metabolically active cells after 1 h incubation at 37 °C under 5% CO_2_ in a humidity incubator. MTT is reduced to purple formazan in the mitochondria of living cells. Formazan crystals in the wells were solubilized in 200 μL of DMSO. After incubation, cell concentration was estimated as ranging from ~5 × 10^4^ to 5.5 × 10^4^ cells per well. Finally, absorbance was measured at 570 nm wavelengths, employing a microplate reader Synergy HT (BioTek Instruments Inc., Winooski, VT, USA). Cell viability was calculated considering controls containing the serum-free medium as 100% viable cells, and using the following formula:

Cell viability (%) = (absorbance of sample)/(absorbance of control) × 100

Data represent the mean and standard deviation (SD) of triplicates of three independent experiments (*n* = 9).

### 2.7. Antioxidant Activity of YE and Its Fractions against Intracellular Reactive Oxygen Species (ROS) Production on H. pylori-Infected Gastric Cells

Intracellular ROS were measured by the DCFH-DA (carboxy-2′,7′-dichloro-dihydro-fluorescein diacetate) (Merck) assay, as previously reported by Silvan et al. [10]. Cells were seeded (~5 × 10^4^ cells per well in 500 μL) in 24-well plates (Sarstedt) and incubated at 37 °C under 5% CO_2_ in a humidity incubator until a monolayer was formed. Cells were incubated with YE and its fractions (YPF and YSF) (0.08 mg/mL) dissolved in a serum-free cell culture medium for 24 h. After that, cells were washed twice with PBS and incubated with 20 mM DCFH-DA (Merck) at 37 °C for 30 min. Next, cells were washed twice with PBS to remove the unabsorbed probe and were then infected with *H*. *pylori* inoculum strains (500 μL) suspended in a serum/antibiotics-free cell culture medium (~1 × 10^8^ CFU/mL). ROS production was immediately monitored for 180 min in a Synergy HT (BioTek Instruments Inc.) fluorescent microplate reader using λ_ex_ 485 nm and λ_em_ 530 nm. After incubation, cell concentration was estimated as ranging from ~5 × 10^5^ to 5.5 × 10^5^ cells per well. After being oxidized by intracellular oxidants, DCFH-DA changes to dichloro-fluorescein (DCF) and emits fluorescence. Cells incubated only with the *H. pylori* inoculum were used as an oxidation control (100% of intracellular ROS production). All samples were analyzed in triplicate in three independent experiments (*n* = 9).

### 2.8. Anti-Inflammatory Activity of YE and Its Fractions on H. pylori-Infected Gastric Cells

The inflammatory response was evaluated as IL-8 production in AGS cells after being infected with different *H. pylori* strains following the procedure described by Silvan et al. [9]. Briefly, human gastric AGS cells were seeded (~5 × 10^4^ cells/well) in 24-well plates (Sarstedt) and incubated in a cell culture medium at 37 °C under 5% CO_2_ in a humidity incubator until a monolayer was formed. Cells were incubated with YE and its fractions (YPF and YSF) (0.08 mg/mL) at 37 °C in a 5% CO_2_ humidified atmosphere for 2 h. Cells were washed twice with PBS and infected with 0.5 mL of *H. pylori* inoculum prepared in a serum/antibiotics-free cell culture medium (~1 × 10^8^ CFU/mL for all tested strains). The infected cells were incubated at 37 °C under 5% CO_2_ for 24 h to allow the bacteria to adhere and invade the cells. Uninfected and nontreated cells were included in the experiment as a negative and positive control of IL-8 production, respectively. At the end of incubation, cell supernatants were collected, particulate material was removed by centrifugation (10 min at 12,000 rpm), and samples were stored at −20 °C until analyses were performed. The amounts of secreted interleukin IL-8 in the collected supernatant from gastric epithelial cell samples were determined by an ELISA assay. A commercially available ELISA kit (Diaclone, Besancon, France) for the quantitation of IL-8 cytokine was used as described by the manufacturer’s instructions. Absorbance was measured at 450 nm using a microplate reader Synergy HT (BioTek Instruments Inc.). Since, in the absence of bacteria, gastric AGS cells release small amounts of IL-8 [42], titers of cytokine released by AGS cells (pg/mL) were determined experimentally. The data represent the mean and SD of triplicates of three independent experiments (*n* = 9).

### 2.9. Antibacterial Activity of YE and Its Fractions against H. pylori Strains

The antibacterial activity of YE and its fractions (YPF and YSF) against the *H. pylori* strains was tested following the procedure described by Silvan et al. [10]. Briefly, 1 mL of the sample at 0.4, 0.2, 0.14, and 0.08 mg/mL (final concentration) was transferred into different flasks containing 4 mL of BB-HS. Bacterial inoculum (100 μL of ~1 × 10^8^ CFU/mL) was then inoculated into the flasks under aseptic conditions. The culture was incubated in the VAIN in the conditions described above. *H. pylori* growth controls were prepared by transferring 100 μL of bacterial inoculum (~1 × 10^8^ CFU/mL) to 5 mL of BB-HS. After 24 h incubation, serial decimal dilutions of cultures were prepared in 0.9% saline solution (NaCl). Then, they were plated onto fresh MHB agar and incubated at 37 °C under microareophillic conditions in the VAIN. After 72 h of incubation, the CFU were assessed. Results were expressed as CFU/mL.

### 2.10. Statistical Analysis

Results were reported as means ± SD. Significant differences among the data were estimated by applying analysis of variance (ANOVA). Tukey’s least significant differences (LSD) test was used to evaluate the significance of the analysis. Differences were considered significant at *p* < 0.05. All statistical tests were performed with IBM SPSS Statistics for Windows, Version 27.0 (IBM Corp., Armonk, NY, USA).

## 3. Results

### 3.1. Characterization of YE and Its Fractions

Phenolic composition of YE and its fractions and the details of HPLC-ESI-QTOF-MS of the identified phenolic compounds are shown in Table 1 and Table S1 (supplementary material). Phenolic compounds from two different families (phenolic acids and flavonoids) were identified in the extract and fractions. Flavonoids were the major family within YE (2924.4 mg/100 g), constituting 77% of the total phenolic compounds. Among the flavonoids, flavones were the prevalent group (2018.1 mg/100 g), representing 69% of flavonoids. Luteolin-7-*O*-glucoside (768.7 mg/100 g; 38% of flavones) and luteolin (447.4 mg/100 g; 22.2% of flavones) were the main compounds identified within YE and in the flavones group.

In the flavonols group (906.3 mg/100 g; 31% of flavonoids), centaureidin and methoxyquercetin isomer were the major compounds accounting for 43% and 41% of total flavonols, respectively. With regards to phenolic acids (22% of total phenolic compounds), chlorogenic acid and its derivatives (1,5-DCQA, 3,4-DCQA, 3,5-DCQA, and 4,5-DCQA) were predominant (75% of total phenolic acids), 3,5-DCQA (361.7 mg/100 g) being the most abundant phenolic acid in YE (43% of total phenolic acids). Rosmarinic acid content was also relevant in YE (22% of total phenolic acids).

Concerning SAF fractions, YPF showed a similar phenolic composition to YE, but it was enriched 2.4 times in total phenolic compounds (9060 mg/100 g) in comparison with YE (3768 mg/100 g). Flavonoid content increases up to 7093.3 (2.4 times more than YE), representing 78% of total phenolic compounds in YPF, similar to that obtained in YE (77%). Within the flavonoids compounds, and as was observed in YE, flavones were the prevalent group (5932.1 mg/100 g), increasing its content up to 83% of total flavonoids, luteolin-7-*O*-glucoside (40% of total flavones) and luteolin (22% of total flavones) being the major compounds in this class of compounds. Phenolic acids concentration increases 2.3 times in YPF compared to YE, chlorogenic acid derivatives being the major compounds in this fraction (98% of total phenolic acids), outstanding the 3,5-DCQA as the most abundant phenolic acid (1163.4 mg/100 g).

On the other hand, only some low-polarity phenolic compounds were recovered as part of YSF (1325.7 mg/100 g), mainly flavonoid compounds (1296.8 mg/100 g) representing 97% of total phenolic compounds identified. Mostly, aglycones of flavonoids, the lesser polar compounds originally described in YE, were found in this fraction. Among them, the biflavonoid amentoflavone (62.2 mg/100 g) and methoxylated flavonols casticin (61.8 mg/100 g) and centaureidin (669.6 mg/100 g) were in significantly (*p* < 0.05) higher concentrations in YSF than in YE. Due to the oleoresin appearance of YSF, it was expected that it could contain volatile oil components. That hypothesis was confirmed with a GC-MS analysis and the results are presented in Table 2.

As shown, a great abundance of monoterpenes and sesquiterpernes was found for both YE and YSF. In particular, four monoterpenes, camphor, artemisia ketone, borneol, and 2,6-dimethyl-1,7-octadiene-3,6-diol, were the most abundant compounds in both extracts. When comparing the total peak area contribution, it can be observed that YSF (43.8 × 10^6^ AUC) represented a double richness of volatile compounds with respect to YE (23.6 × 10^6^ AUC). The fraction obtained in the precipitator vessel (YPF) was also analyzed; however, as expected, it lacks volatile components (data not shown).

### 3.2. Antioxidant Activity of YE and Its Fractions against Intracellular ROS Production in H. pylori-Infected AGS Cells

**Figure 1:**
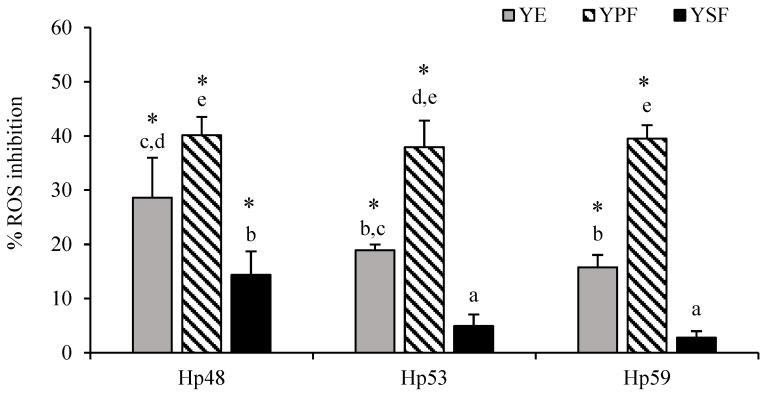
Inhibition effect of yarrow extract (YE) and its fractions (YPF and YSF) (0.08 mg/mL) on ROS production by human gastric epithelial AGS cells after *H. pylori* strains infection. Values are the mean ± SD (*n* = 3). * Asterisk indicates significant differences compared to the untreated infected control (no inhibition) (*p* < 0.05). ^a,b,c,d,e^ Different letters indicate statistical difference between samples and *H. pylori* strains (*p* < 0.05).

Before the antioxidant activity experiments, the viability of the AGS cells was evaluated in the presence of YE and its corresponding fractions (YPF and YSF). For this purpose, AGS cells were placed in contact with variable concentrations of YE and its fractions (0.08 to 0.40 mg/mL), and the MTT assay was performed. The data obtained demonstrated that the maximum concentration that was non-cytotoxic resulted in 0.08 mg/mL for all tested samples (>95% cell viability) (data not shown). Higher concentrations of YE and its fractions significantly reduced cell viability compared to the control of untreated cells (viability lower than 80%). Infection of gastric cells with *H. pylori* strains (Hp48, Hp53, and Hp59) induced ROS production in AGS cells (data not shown), as has been demonstrated in previous works using this cell model [10]. As shown in Figure 1, in all cases, YE and YPF significantly (*p* < 0.05) reduced intracellular ROS production in AGS-infected cells in comparison with the control group (untreated infected cells). However, YSF had a strain-dependent behavior and only significantly inhibited (*p* < 0.05) ROS production when AGS cells were infected with the Hp48 strain.

The inhibition effect of YE on ROS production ranged from 16% to 29% depending on the *H. pylori* strain. YPF, the fraction enriched in phenolic compounds, was the most active fraction regardless of the strain used. It provoked the inhibition of intracellular ROS production of about 40%. YSF, which contained only the most non-polar phenolic compounds, showed a lower antioxidant activity (3–14%) than the YE, which is also coherent with the presence of lower amounts of phenolic compounds in this fraction.

### 3.3. Effect of the YE and Its Fractions on the Inflammatory Response Induced by H. pylori in AGS Cells

**Figure 2:**
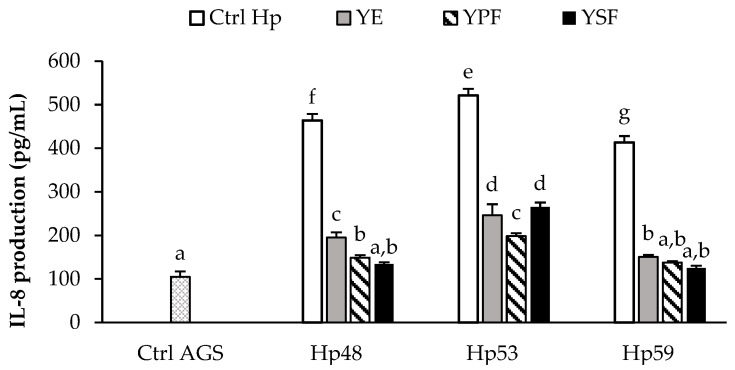
Inhibition effect of yarrow extract (YE) and its SAF fractions (YPF, yarrow’s precipitator fraction and YSF, and yarrow’s separator fraction) (0.08 mg/mL) on pro-inflammatory cytokine IL-8 production (pg/mL) by human gastric epithelial AGS cells infected by *H. pylori* strains. Control Hp (Ctrl Hp) represents the values obtained from untreated cells infected with *H. pylori* strains. Control AGS (Ctrl AGS) represents the values obtained from untreated and uninfected AGS cells. Values are the mean ± SD (*n* = 3). ^a–g^ Different letters indicate statistical differences between treatments for each *H. pylori* strain (*p* < 0.05).

Previously, we evaluated in vitro the secretion of different pro-inflammatory cytokines produced in *H. pylori*-infected AGS cells, IL-8 being the most secreted cytokine, similarly to that described by others [2]. For this reason, we selected IL-8 as a biomarker to evaluate the anti-inflammatory effect of YE and its fractions on AGS cells infected by *H. pylori* strains. As can be observed in Figure 2, the background level of IL-8 production in uninfected AGS cells was 105.0 ± 12.0 pg/mL (Ctrl. AGS; untreated and uninfected cells). Infection with *H. pylori* strains effectively stimulated the secretion of IL-8 pro-inflammatory cytokine (Ctrl. Hp; untreated infected control) in AGS cells (413 to 521 pg/mL). Furthermore, IL-8 production showed a strain-dependent character, since statistical differences between strains were found (*p* < 0.05).

For all strains, YE significantly (*p* < 0.05) decreased IL-8 production by 53% to 64% when compared to its respective control Hp. Unlike the antioxidant activity, it was more difficult in this case to evaluate the impact of each fraction on the observed behavior. For two of the strains (Hp48 and Hp59), both the fraction enriched in phenolic compounds (YPF) and the fraction containing essential oils (YSF) showed similar behaviors, reducing IL-8 production, suggesting that the two types of compounds could contribute to IL-8 inhibition. In contrast, for the Hp53 strain, YPF presented a greater contribution to IL-8 inhibition than YSF (*p* < 0.05).

### 3.4. Antibacterial Activity of YE and Its Fractions against H. pylori Strains

The antibacterial effect of YE, YPF, and YSF against *H. pylori* growth is presented in Table 3. YE was significantly (*p* < 0.05) effective as an antibacterial agent against all *H. pylori* strains tested, although the effect was greater or lesser depending on the strain and varied in a range of CFU reduction between 4.8 and 7.1 log. However, MIC was the same for all strains (0.14 mg/mL). Analysis of the contribution of each fraction to the antibacterial effect showed that YSF, the fraction enriched in volatile compounds, had a significantly (*p* < 0.05) greater antibacterial effect (6.3–7.1 log CFU reduction, depending on the strain) and lower MIC (0.08 mg/mL) than YE. On the other hand, phenolic-enriched YPF also significantly (*p* < 0.05) reduced bacterial growth of all strains and this reduction was independent of the strain used.

## 4. Discussion

The phenolic composition of the YE obtained by ethanolic extraction was similar to that reported in previous works for this same yarrow variety [26,35]. The use of ethanol or ethanol mixtures as extraction solvents has been described as a useful method to obtain extracts rich in bioactive phenolic compounds and volatile essential oils from yarrow [26,43]. Because of the well-known bioactivities of phenolic compounds contained in yarrow, the SAF technique was employed to selectively obtain enriched fractions from YE, according to its greater or lesser affinity to the SC-CO_2_ and ethanol mixture performing as solvents. YPF was enriched in phenolic compounds, while YSF was enriched in monoterpenes and sesquiterpenes, which are very abundant compounds in yarrow’s essential oil [13,19]. It has been described that these fractions represent an advantage in the recovering of the extract with high purity and free of solvent, contributing to producing high-quality products [36]. YE and its fractions (YPF and YSF) demonstrated their potential utility for use in both the control of *H. pylori* growth and the modulation of the oxidative and inflammatory response of the human gastric cells associated with *H. pylori* infection. Modulation of the oxidative and inflammatory response in the gastric epithelium has been shown to be particularly relevant in preventing tissue damage and the progression of pathologies associated with *H. pylori* infection [2]. YPF, which presents phenolic compounds 2.4 times more concentrated than YE, had the highest inhibitory activity for ROS production. This behavior seems consistent with the potent antioxidant activity described for many of the major phenolic compounds identified in this fraction. For example, the flavones luteolin-7-*O*-glucoside and luteolin, the most predominant phenolic compounds in the YPF fraction, have been described as potent antioxidant agents, since their molecular structure, formed by a 2–3 carbon double bond of C ring (C2=C3) conjugated with a carbonyl group in C4, confers them with the capacity to react and neutralize ROS, behaving as scavengers in the cellular processes that generate this type of molecules [44]. Other major compounds in YE concentrated in the YPF, such as 3,5-DCQA, have also been shown to have a relevant capacity to scavenge intracellular ROS [45]. In general, since in YPF most of the phenolic compounds present are in a higher concentration than in YE, it is expected that many of them, whose antioxidant properties have been described [46,47,48], may contribute to a higher inhibition of ROS production found for YPF. On the other hand, the scarce presence of phenolic compounds in the YSF fraction was correlated with low antioxidant activity. The high antioxidant capacity of phenolic-compound-enriched YPF also led to a decrease in IL-8 production. In the case of YE and YSF, not only phenolic compounds but also some essential oils seemed to be involved in their anti-inflammatory capacity. Similar results have recently been obtained evaluating the effect of a yarrow extract and its fractions on differentiated human macrophages, observing that the inhibition in the secretion of some pro-inflammatory cytokines (IL-6, IL-1*β*, and TNF-α) could be related to the presence of essential oils such as camphor, borneol, or artemisia ketone, which constituted approximately 30% of the fraction studied [37]. Likewise, luteolin-7-*O*-glucoside and luteolin, predominant phenolic compounds in the YE and YPF, have been shown to be able to downregulate IL-1*β*, IL-6, and TNF-*α* production acting on NF-κB, MAPK, and JAK/STAT inflammatory pathways by reducing inflammation in cellular models [44,49]. It has also been reported in experiments carried out with a hydro-alcoholic extract of thistle that 3,5-dicaffeoylquinic acid, another major phenolic compound in YE and YPF, was primarily responsible for inhibiting the secretion of IL-8 and NF-κB pathways in human gastric epithelial AGS cells [50].

Although YE and its two fractions were effective as inhibitors of *H. pylori* growth, the contribution of YSF was higher in the antibacterial activity of the extracts. Numerous essential oils are known to have significant antibacterial activity against *H. pylori* [51]. Particularly in yarrow, the major volatile compounds identified (camphor, borneol, and artemisia ketone) have also been shown to be effective as inhibitors of *H. pylori* growth [48,49,50,51,52,53,54]. Although the *H. pylori* strain may influence the intensity of the bioactive response obtained, the present work showed that YE and its fractions were effective as antioxidant, anti-inflammatory, and antibacterial agents regardless of the characteristics of the used strain.

## 5. Conclusions

Among other uses, yarrow has been widely utilized as a part of folk medicine to alleviate symptoms related to gastrointestinal discomfort, many of them similar to those associated with *H. pylori* infection. The historical background of its efficacy in the treatment of these pathologies is complemented in this work by more scientifically based evidence to support the pharmacological effects of various compounds present in YE against *H*. *pylori*. YE may be potentially effective in combating oxidative stress and modulating the inflammatory response associated with gastric *H. pylori* infection. In addition, YE exhibits strong antibacterial activity against *H. pylori*. Both the phenolic compounds and essential oils present in the extract appear to contribute to the bioactive properties of the extract, although the degree of contribution varies depending on each property (antioxidant, anti-inflammatory, or antibacterial). The SAF technique allows the obtaining of YE fractions enriched in phenolic compounds or essential oils, on the basis of the concept of green extraction, and may be useful in the design of bioactive extracts against *H. pylori* in which it is desirable to enhance specific bioactivity. This approach is attractive in terms of cost, tolerability, and cultural acceptability and can be especially useful in those countries where modern health facilities and access to certain pharmacological substances are not always adequate or available.

## Figures and Tables

**Table 1:** Phenolic composition and quantification of YE and its fractions (mg/100 g dry sample).

Phenolic Compounds	YE	YPF	YSF
*Phenolic acids*			
*Hydroxycinnamic acids*			
Caffeic acid ^1^	17.4 ± 0.1 ^a^	-	18.4 ± 0.1 ^b^
Caftaric acid ^1^	<L.Q.	22.5 ± 0.3	-
Chlorogenic acid ^1^	61.7 ± 0.2 ^a^	190.7 ± 4.1 ^b^	-
Cryptochlorogenic acid ^1^	1.1 ± 0.1 ^a^	4.4 ± 1.4 ^a^	-
1,5- DCQA ^1^	68.7 ± 0.7 ^a^	179.7 ± 8.7 ^b^	-
3,4- DCQA ^1^	38.3 ± 5.1 ^a^	69.1 ± 0.5 ^b^	-
3,5- DCQA ^1^	361.7 ± 1.8 ^b^	1163.4 ± 10.2 ^c^	10.4 ± 0.1 ^a^
4,5- DCQA ^1^	96.6 ± 0.9 ^a^	318.7 ± 0.2 ^b^	-
Ferulic acid ^1^	7.9 ± 1.7 ^b^	4.3 ± 0.1 ^a^	-
Neochlorogenic acid ^1^	5.8 ± 0.1 ^a^	13.8 ± 0.14 ^b^	-
Rosmarinic acid ^1^	185.0 ± 1.2	-	-
*Σ Total Phenolic acids*	844.2 ^b^	1966.6 ^c^	28.8 ^a^
*Flavonoids*			
*Flavones*			
Amentoflavone	41.9 ± 0.1 ^a^	41.2 ± 0.1 ^a^	62.2 ± 0.1 ^b^
Apigenin ^1^	195.7 ± 0.4 ^b^	474.2 ± 0.4 ^c^	92.5 ± 0.1 ^a^
Apigenin-*C*-hexoside-*C*-pentoside	30.3 ± 0.1 ^a^	84.7 ± 0.2 ^b^	-
Apigenin-7-*O*-glucoside ^1^	179.5 ± 0.8 ^a^	587.7 ± 1.3 ^b^	-
Diosmetin ^1^	50.1 ± 0.1 ^a^	72.8 ± 0.1 ^b^	50.2 ± 0.1 ^a^
Homoorientin ^1^	2.5 ± 0.7 ^a^	15.5 ± 0.1 ^b^	-
6-Hydroxyluteolin-7-*O*-glucoside	145.2 ± 0.7 ^a^	466.2 ± 0.4 ^b^	-
Luteolin ^1^	447.4 ± 1.2 ^b^	1304.0 ± 10.0 ^c^	95.5 ± 1.2 ^a^
Luteolin-6,8-di-*C*-glucoside	46.9 ± 0.2 ^a^	151.1 ± 0.1 ^b^	-
Luteolin-7-*β*-glucuronide ^1^	19.9 ± 1.1 ^a^	59.3 ± 2.8 ^b^	-
Luteolin-7-*O*-glucoside ^1^	768.7 ± 8.0 ^a^	2385.3 ± 97.5 ^b^	-
Schaftoside ^1^	27.2 ± 0.1 ^a^	88.8 ± 0.4 ^b^	-
Schaftoside isomer	26.2 ± 0.3 ^a^	89.6 ± 0.2 ^b^	-
Vicenin 2 ^1^	36.6 ± 0.5 ^a^	111.7 ± 0.6 ^b^	-
*Σ Total Flavones*	2018.1 ^b^	5932.1 ^c^	300.4 ^a^
*Flavonols*			
Casticin ^1^	28.6 ± 0.1 ^a^	-	61.8 ± 0.6 ^b^
Centaureidin	391.3 ± 0.4 ^a^	107.8 ± 0.1 ^b^	669.6 ± 0.4 ^c^
Methoxyquercetin isomer	376.0 ± 0.8 ^b^	751.3 ± 0.8 ^c^	265.0 ± 0.2 ^a^
Quercetin ^1^	47.0 ± 0.1 ^a^	143.6 ± 0.8 ^b^	-
Rutin ^1^	50.6 ± 1.0 ^a^	133.7 ± 2.2 ^b^	-
Vitexin ^1^	12.8 ± 0.8 ^a^	24.8 ± 0.7 ^b^	-
*Σ Total Flavonols*	906.3 ^a^	1161.2 ^c^	996.4 ^b^
*Σ Total Flavonoids*	2924.4 ^b^	7093.3 ^c^	1296.8 ^a^
*Σ Total phenolic compounds*	3768.6 ^b^	9060.0 ^c^	1325.7 ^a^

YE: yarrow extract. YPF: yarrow’s precipitator fraction. YSF: yarrow’s separator fraction. <L.Q.: below limit of quantification. ^1^ Comparison with authentic standard. ^a,b,c^ Values in the same row marked with different superscript letters indicates statistical differences (*p* < 0.05).

**Table 2:** Volatile compounds identified by GC-MS in YE and YSF represented as peak area contributions (normalized percentage of area).

Rt	Compound	YE	YSF
11.9	Yomogi alcohol	2.5	2.7
13.1	Eucalyptol	5.2	4.2
13.5	γ-Vinyl-γ-valerolactone	2.1	1.8
14.6	Artemisia ketone	13.3	11.5
18.4	Camphor	16.7	15.0
19.5	Borneol	10.5	10.5
20.7	3,7-dimethyl-1,5-Octadiene-3,7-diol	7.1	7.5
22.8	trans-Chrysanthenyl acetate	2.9	3.0
24.3	(5E)-5,9-dimethyl-5,8-decadien-2-one	1.8	1.6
24.7	2,6-dimethyl-1,7-octadiene-3,6-diol	10.5	11.3
26.0	N.i.	8.7	10.4
30.3	Jasmone	1.9	1.8
31.2	β-Caryophyllene	1.7	1.9
38.2	β-Caryophyllene oxide	6.0	7.0
41.7	β-Eudesmol	1.5	1.9
51.8	Saussurea lactone	4.1	3.9
52.8	Hexahydrofarnesyl acetone	3.6	4.2
	ƩAUC	23.6 × 10^6^	43.8 × 10^6^

Rt: retention time. YE: yarrow extract. YSF: yarrow’s separator fraction. N.i.: non-identified compound. AUC: area under curve.

**Table 3:** Antibacterial activity of YE and its fractions (YPF and YSF) at 0.4 mg/mL against *H. pylori* strains. Results represent the mean ± standard deviation of colony forming units (CFU)/mL (*n* = 3).

		YE			YPF			YSF		
Strains	Control Growth	CFU/mL	Log CFU Reduction	MIC (mg/mL)	CFU/mL	Log CFU Reduction	MIC (mg/mL)	CFU/mL	Log CFU Reduction	MIC (mg/mL)
Hp48	3.6 ± 0.3 × 10^8^ _c,A_	<1.0 × 10^2^ _a,A_	>6.3	0.14	4.0 ± 0.7 × 10^5^ _b,A_	2.4	0.08	<1.0 × 10^2^ _A,a_	>6.3	0.08
Hp53	3.4 ± 1.6 × 10^8^ _d,A_	5.0 ± 1.5 × 10^3^ _b,B_	4.8	0.14	6.5 ± 1.3 × 10^5^ _c,A_	2.2	0.14	<1.0 × 10^2^ _a,A_	>6.8	0.08
Hp59	9.9 ± 0.9 × 10^8^ _d,A_	1.4 ± 0.2 × 10^2^ _b,A_	7.1	0.14	4.8 ± 1.6 × 10^6^ _c,B_	2.4	0.14	<1.0 × 10^2^ _a,A_	>7.1	0.08

CFU detection limit was 1.00 × 10^2^ CFU/mL. MIC: minimal inhibitory concentration (mg/mL). YE: yarrow extract. YPF: yarrow’s precipitator fraction. YSF: yarrow´s separator fraction. ^a,b,c,d^ Different lowercase letters denote significant differences within a line (*p* < 0.05). ^A,B^ Different uppercase letters denote significant differences within a column (*p* < 0.05).

## Data Availability

The data presented in this study are available in this manuscript.

## Supplementary Materials

The following supporting information can be downloaded at: https://www.mdpi.com/article/10.3390/antiox11101849/s1, Table S1: Phenolic compounds identified in yarrow samples by using HPLC-ESI-QTOF-MS.
