# Correction: Villalva et al. Antioxidant, Anti-Inflammatory, and Antibacterial Properties of an *Achillea millefolium* L. Extract and Its Fractions Obtained by Supercritical Anti-Solvent Fractionation against *Helicobacter pylori*. *Antioxidants* 2022, *11*, 1849

**DOI:** 10.3390/antiox13030306

**Published:** 2024-02-29

**Authors:** Marisol Villalva, Jose Manuel Silvan, Teresa Alarcón-Cavero, David Villanueva-Bermejo, Laura Jaime, Susana Santoyo, Adolfo J. Martinez-Rodriguez

**Affiliations:** 1 Microbiology and Food Biocatalysis Group (MICROBIO), Department of Biotechnology and Food Microbiology, Institute of Food Science Research (CIAL, CSIC-UAM), C/Nicolás Cabrera, 9. Cantoblanco Campus, Autonomous University of Madrid, 28049 Madrid, Spain; marisol.villalva@csic.es (M.V.); jm.silvan@csic.es (J.M.S.); 2 Microbiology Department, Hospital Universitario de La Princesa, 28006 Madrid, Spain; 3 Department of Preventive Medicine, Public Health and Microbiology, School of Medicine, Autonomous University of Madrid, 28029 Madrid, Spain; 4 Department of Production and Characterization of Novel Foods, Institute of Food Science Research (CIAL, CSIC-UAM), C/Nicolas Cabrera, 9. Cantoblanco Campus, Autonomous University of Madrid, 28049 Madrid, Spainlaura.jaime@uam.es (L.J.);

Table S1 in the Supplementary Material (Phenolic compounds identified in yarrow samples by using HPLC-ESI-QTOF-MS) in the original paper [1] was updated after a revision of the chromatographic identification. The update adds additional information, which affects some of the previously reported MS/MS ions (*m*/*z*), as some of them were omitted (e.g., vicenin 2), contained typographical errors (e.g., apigenin, diosmetin), or included an inaccurate *m*/*z* or abundance of the detected ions (e.g., amentoflavone, apigenin-*C*-hexoside-*C*-pentoside, chlorogenic acid, cryptochlorogenic acid, schaftoside, schaftoside isomer). In addition, the classification of vitexin was inappropriate and was updated (moved from flavonols to the flavones section), as was the accurate mass (*m*/*z*) of ferulic acid, which was reported with a typographical error.

The authors state that the changes made only affect the results shown in Table S1 and enhance the information given therein, without affecting in any way the scientific conclusions of the published work.

The authors would like to thank Franski et al. for their comments, which have allowed us to correct these minor issues in Table S1. Table S1 was updated on 4 July 2023.
