# Supplementary material for: Antioxidant, Anti-Inflammatory, and Antibacterial Properties of an Achillea millefolium L. Extract and Its Fractions Obtained by Supercritical Anti-Solvent Fractionation against Helicobacter pylori
Source: Antioxidants (Basel). 2022 Sep 20;11(10):1849. doi: 10.3390/antiox11101849 (PMC9598488; doi:10.3390/antiox11101849)
Supplement: Supplementary file 1 [file antioxidants-11-01849-s001.zip › antioxidants-1896162-supplementary.pdf]

Table S1. Phenolic compounds identified in yarrow samples by using HPLC-ESI-QTOF-MS.

Phenolic compounds	Rt (min)	Theoretical mass (*m/z*)	Accurate mass (*m/z*)	MS/MS ions (*m/z*)
Phenolic acids				
Hydroxycinnamic acids				
Caffeic acid [1]	18.0	179.0350	179.0353	135 (100)
Caftaric acid [1]	13.8	311.0409	311.0410	179 (14), 149 (100)
Chlorogenic acid [1]	15.0	353.0878	353.0877	191 (100), 161 (10)
Cryptochlorogenic acid [1]	15.3	353.0878	353.0877	191 (100), 161 (11)
1,5- DCQA[1]	26.7	515.1195	515.1190	353 (100), 191 (40)
3,4- DCQA [1]	25.6	515.1195	515.1189	353 (100), 335 (30), 179 (69), 173 (80)
3,5- DCQA [1]	27.0	515.1195	515.1190	353 (100), 191 (55), 179 (35), 135 (21)
4,5- DCQA [1]	28.4	515.1195	515.1190	353 (100), 191 (10), 179 (30), 173 (40)
Ferulic acid [1]	25.0	193.0510	103.0504	178 (50), 134 (100)
Neochlorogenic acid [1]	13.0	353.0878	353.0877	191 (100), 179 (76), 135 (40)
Rosmarinic acid [1]	28.9	359.0772	359.0771	197 (80), 179 (50), 161 (100), 135 (30)
Flavonoids				
Flavones				
Amentoflavone	39.5	537.0900	537.0821	519.0716 (50), 495.0721 (10)
Apigenin [1]	37.2	269.0455	269.0454	112 (100)
Apigenin-*C*-hexoside-*C*-pentoside	19.5	563.1406	563.1401	473 (10), 443 (20)
Apigenin-7-*O*-glucoside [1]	27.8	431.0984	431.0980	269 (100)
Diosmetin [1]	37.8	299.0561	299.0554	112 (100)
Homoorientin [1]	18.9	447.0933	447.0930	429 (30), 357 (100), 327 (80)
6-Hydroxyluteolin- 7-*O*-glucoside	20.0	463.0882	463.0880	301 (100)
Luteolin [1]	33.8	285.0405	285.0400	175 (80), 151 (100), 107 (51)
Luteolin-6,8-di-*C*-glucoside	19.7	609.1461	609.1453	489 (100), 325 (40)
Luteolin-7-*β*-glucuronide [1]	24.1	461.0725	461.0722	285 (100)
Luteolin-7-*O*-glucoside [1]	23.8	447.0933	447.0928	285 (100)
Schaftoside [1]	18.4	563.1406	563.1401	473 (10), 443 (20)
Schaftoside isomer	18.2	563.1406	563.1401	473 (10), 443 (20)
Vicenin 2 [1]	16.0			
Flavonols				
Casticin [1]	45.7	373.0929	373.0923	358 (43), 343 (90)
Centaureidin	40.1	359.0772	359.0770	344 (59), 229 (100)
Methoxyquercetin isomer	35.4	315.0510	315.0508	301 (100)
Quercetin [1]	34.1	301.0354	301.0352	151 (60)
Rutin [1]	22.1	609.1097	609.1093	301 (100)
Vitexin [1]	22.4	431.0984	431.0981	311 (100)

Rt, retention time. [1]Comparison against its authentic standard.
